# Optimal-Enhanced Solar Cell Ultra-thinning with Broadband Nanophotonic Light Capture

**DOI:** 10.1016/j.isci.2018.04.018

**Published:** 2018-04-26

**Authors:** Manuel J. Mendes, Sirazul Haque, Olalla Sanchez-Sobrado, Andreia Araújo, Hugo Águas, Elvira Fortunato, Rodrigo Martins

**Affiliations:** 1 i3N/CENIMAT, Department of Materials Science, Faculty of Science and Technology, Universidade NOVA de Lisboa and CEMOP/UNINOVA, Campus de Caparica, 2829-516 Caparica, Portugal

**Keywords:** Optical Materials, Computational Method in Materials Science, Energy Materials

## Abstract

Recent trends in photovoltaics demand ever-thin solar cells to allow deployment in consumer-oriented products requiring low-cost and mechanically flexible devices. For this, nanophotonic elements in the wave-optics regime are highly promising, as they capture and trap light in the cells' absorber, enabling its thickness reduction while improving its efficiency. Here, novel wavelength-sized photonic structures were computationally optimized toward maximum broadband light absorption. Thin-film silicon cells were the test bed to determine the best performing parameters and study their optical effects. Pronounced photocurrent enhancements, up to 37%, 27%, and 48%, respectively, in ultra-thin (100- and 300-nm-thick) amorphous, and thin (1.5-μm) crystalline silicon cells are demonstrated with honeycomb arrays of semi-spheroidal dome or void-like elements patterned on the cells' front. Also importantly, key advantages in the electrical performance are anticipated, since the photonic nano/micro-nanostructures do not increase the cell roughness, therefore not contributing to recombination, which is a crucial drawback in state-of-the-art light-trapping approaches.

## Highlights

• Wave-optics allows broadband light trapping while minimizing solar cell thickness
• Photonic transparent electrodes can boost both optical and electrical performance
• Up to 48% efficiency enhancement is determined relative to conventional approaches
• Performance enhancement is demonstrated for a wide incidence angular range (0°–70°)

## Graphical Abstract

**Figure:**
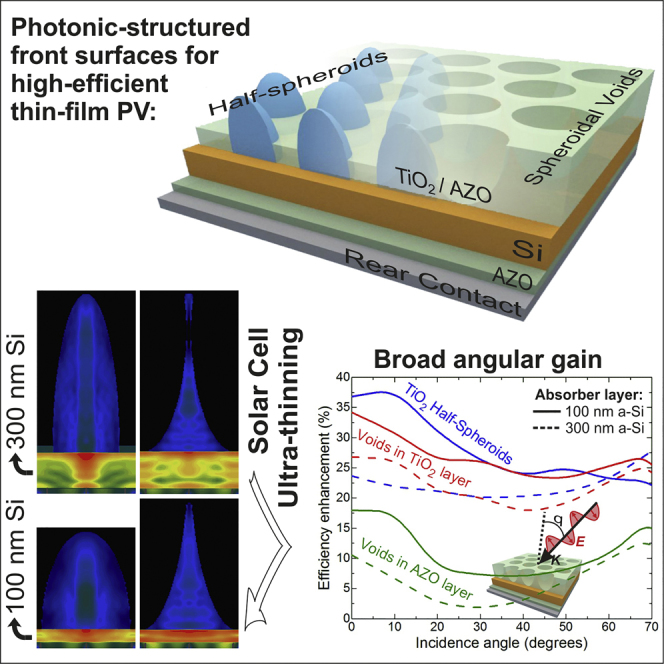

## Introduction

The application of light trapping (LT) solutions in photovoltaic (PV) devices is considered the most promising route to reduce their thickness while improving the sunlight-to-electricity conversion efficiency. LT has been mainly applied to both reduce reflection and boost light absorption in solar cells, allowing thickness reduction while enhancing the generated photocurrent (Polman and Atwater, 2012). Optically thicker but physically thinner cells imply cheaper and faster fabrication, lightweight, and improved flexibility (Lin et al., 2014, Vicente et al., 2018). Besides, thickness reduction can lead to higher open-circuit voltages (and consequently efficiencies) due to lower bulk recombination (Polman et al., 2016).

The LT approaches conventionally applied in wafer-based devices rely on textured rear/front surfaces, which provide anti-reflection, via geometrical refractive index matching provided by the front facets, together with light scattering, which increases absorption via optical path-length amplification within the absorber layer (Priolo et al., 2014, Andreani et al., 2015). Optimized texturing has shown absorption enhancements in crystalline silicon (c-Si) wafers close to the fundamental *4n*^*2*^ LT limit of geometrical optics (Ingenito et al., 2014). However, when applied in thin-film cells, the textures' size must be reduced along with the absorber thickness, which lowers their LT effects (Haug and Ballif, 2015, Branham et al., 2016). Nonetheless, the main drawback of texturing is that it increases the roughness (hence surface area) and defect density in the PV material, which deteriorate the cells' electrical transport via the increase of charge carrier trapping and recombination. Several alternative strategies have been investigated for thin-film PV, employing nano/micro-structures with dimensions comparable to or smaller than the illuminating wavelengths such as diffraction gratings (Mellor et al., 2011, Schuster et al., 2015), micro-lenses (Mendes et al., 2010, Grandidier et al., 2012, Yang et al., 2016), Mie features (Spinelli et al., 2012, Spinelli and Polman, 2014, Zhou et al., 2014, Van Lare et al., 2015), and plasmonic nanoparticles (Mendes et al., 2014, Mendes et al., 2015, Morawiec et al., 2014). However, many of these alternative approaches also require structuring the PV layers, thus suffering from the same electrical compromise of texturing, and none has yet led to efficiencies superior to those attained with optimized periodic texturing, as applied in record-efficient (13.6%) thin-film Si cells (Sai et al., 2015).

At present, the use of high-refractive-index dielectric front structures with wavelength-scale features is considered the preferential approach to attain maximum LT in thin-film PV without deteriorating the cells' electrical performance (Tseng et al., 2012, Li et al., 2013, Brongersma et al., 2014, Yang et al., 2016, Sanchez-Sobrado et al., 2017). Such nanostructures operate in the complex regime of wave-optics, where interference-related optical mechanisms contribute to LT. Therefore it is essential to perform optimizations of the full set of physical parameters, employing exact electromagnetic formalisms as in this work, to determine the best performing materials and geometries. For instance, optimized hexagonal arrays of TiO_2_ half-spheroids, integrated in the cell front, can allow 43.3% current enhancement relative to optimized anti-reflection coatings (ARCs) (Mendes et al., 2016). The key advantages of this type of front-located dielectric nanophotonic elements, relative to other LT approaches, are the following. Optically, their combined light incoupling and confinement effects can provide broadband photocurrent enhancement in different portions of the spectrum. This is due to their dome/cone-like shape providing effective index matching with the high-index absorber layer, which can almost eliminate reflection at short wavelengths (in ultraviolet-visible [UV-Vis]) above the PV material bandgap. At the same time, their strong forward scattering leads to absorption enhancement at the longer near-infrared (NIR) wavelengths close to the bandgap, via light focusing in the intense near field generated beneath the particles and path-length amplification of the propagating far field (Mendes et al., 2011). Such optical properties can be tuned by adjusting the particles' geometry, thus allowing their customization for different types of PV devices. For instance, shapes with higher aspect ratio exhibit stronger anti-reflection, whereas lower aspect ratio enables more effective light scattering and coupling to wave-guided modes. Electrically, they can be incorporated in the top (front surface) transparent conductive oxide (TCO) of completed cells with flat layers. Thus the structures neither increase the roughness nor the surface area of the cell layers and therefore do not degrade the cells' electric performance via increase of carrier recombination. The front location of the photonic elements is allowed by their optically lossless dielectric material, in the relevant spectral range, which provides little parasitic absorption. Mechanically, the arrays of photonic elements are not expected to have detrimental effects on the bendability of thin-film cells, relative to flat ARC layers (Yu et al., 2013), enabling their integration on flexible platforms.

In this work, such LT strategy is advanced in three ways. First, the design of the photonic structures is developed, considering front-located elements with inverse geometries composed of semi-spheroidal void arrays in the photonic media, which can provide higher degrees of light spreading within the cell absorber layer and can be more easily fabricated by high-throughput soft lithography methods. Second, besides high-index dielectric materials, the void nanostructures are developed with TCO (i.e., aluminium zinc oxide [AZO]) media that can improve the cells' front contact while providing LT. The enhancements obtained with the TiO_2_ dielectric structures are higher than those made of AZO, due to the higher real part and lower imaginary part (associated to parasitic absorption) of TiO_2_. Nevertheless, the AZO photonic coatings offer a key electrical advantage since they enable the use of higher TCO volumes in the cells' front contact, which decreases its sheet resistance while improving its optical properties. Third, the photonic nanostructures were designed to achieve maximum broadband absorption in different Si layers, namely, in amorphous silicon (a-Si) with 100 and 300 nm thickness and in 1,500-nm-thick c-Si, attaining pronounced photocurrent gains (up to 37%, 27%, and 48%, respectively). This is highly relevant to minimize the cells' thickness with minimal efficiency loss, thereby improving the cells' mechanical bendability and facilitating its conformal integration in various flexible substrates (e.g., polymers/plastics [Yu et al., 2013, Nam et al., 2016], fabrics [Qiu et al., 2014], and paper-based substrates [Águas et al., 2015, Vicente et al., 2017b]) of consumer items such as wearables, smart packaging/labeling, curved surfaces in buildings or vehicles, internet-of-things systems, and mobile medical testing. In addition, flexible cells offer important cost savings to PV industry, as they can be manufactured in large-area roll-to-roll processes and have reduced material costs due to their small thickness and lower installation costs.

A multi-parameter optimization algorithm, implemented in an electromagnetic finite-difference time-domain (FDTD) program, is used to determine the parameters of such structures that provide the highest photocurrent enhancement in thin-film Si cells. The results are compared with the theoretical LT limits of geometrical optics, achieved with idealized *Lambertian* scattering surfaces (Bozzola et al., 2012, Andreani et al., 2015). Such limits neglect light interference effects and therefore are not applicable to the present cell structures with wavelength-sized features and absorber thicknesses. However, they are useful to compare, for gaining a deeper understanding of the underlying interference-related mechanisms that contribute to LT in our structures in the wave-optics regime (Callahan et al., 2012).

The optimizations were conducted for illumination normal to the cells. Therefore another important aspect analyzed here is how the broadband LT effects are affected for different incidence angles. It was found that even though the resulting photonic-enhanced current tends to decrease with increasing angle, it remains significantly above that provided by the reference ARC layer for all angles.

Thin-film Si cells were the test bed for the light management solutions presented here, but they can be readily implemented in any other type of PV devices. The applicability of our LT schemes is facilitated by the fact that they are not embedded in the cell layer structure and can be incorporated as a final processing step on top of already-completed cells with a substrate-type layer configuration (termed *n-i-p* in Si-based devices). Advantageously, such configuration is compatible with nearly all types of thin-film cells, particularly those requiring high-temperature processing such as the copper indium gallium (di)selenide (CIGS [Van Lare et al., 2015, Yin et al., 2016]) and with the aforementioned flexible applications that typically use opaque bendable substrates (Haug and Ballif, 2015).

## Results

**Figure 1:**
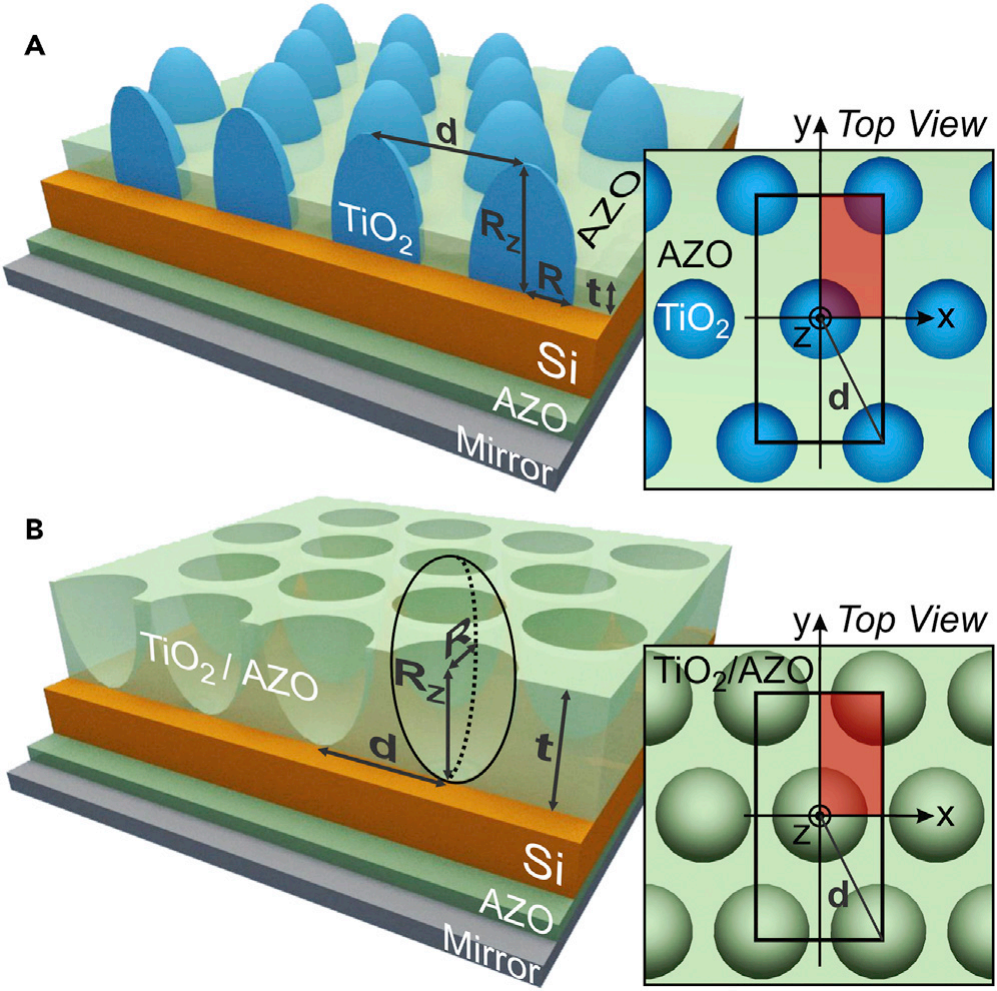
Sketches of Both Types of LT Nanostructures Analyzed in This Work, Patterned on Solar Cells with a Si Absorber (either a-Si or c-Si) (A and B) The LT elements provide a gradually varying effective refractive index, which minimizes reflection, while their geometry can interact with the incoming light to produce strong scattered fields preferentially directed into the higher index Si material with high mode density. The parameters (*R, R*_*Z*_*, t, d*) considered for optimization are indicated by the arrows. The LT structures consist in an hexagonal array (with pitch *d*) of vertically aligned spheroidal-based features with radii *R* and *R*_*Z*_, respectively, along the in-plane and normal directions. (A) TiO_2_ half-prolates separated by an AZO layer with thickness *t*; (B) semi-prolate voids in a layer of either TiO_2_ or AZO material with thickness *t*. At normal incidence, light impinges from the top along the spheroids' axis of revolution (*z*). The rear side of the Si layer is coated with a 60-nm-thick passivating AZO layer and a perfect reflecting mirror. The sketches on the right show the top views of the structures, whose symmetry allows the computed region to be reduced to the volume in red. See Section S1 in Supplemental Information for further details regarding the computational methods.

A numerical mesh-based FDTD formalism (Lumerical Solutions, Inc., 2017) was used to model the 3D electric field distribution produced in the two types of structures shown in Figure 1. This is a widely employed method to calculate the optical response of thin-film solar cells with photonic structures, having arbitrary materials and geometries (Grandidier et al., 2011, Brongersma et al., 2014, Branham et al., 2016, Mendes et al., 2016, Sanchez-Sobrado et al., 2017). The details of the computational method are given in Section S1 of Supplemental Information.

A key technological advantage offered by LT is the possibility of lowering the solar cells' costs, since it allows reducing the absorber thickness without efficiency loss. Nevertheless, this only makes sense if the LT structures can be fabricated by large-scale inexpensive processes, compatible with PV industry, that do not imply a significant increase in the devices' manufacturing price (Polman et al., 2016). For this, scalable soft-lithography techniques, such as nano-imprint (Yin et al., 2016) or colloidal lithography (CL) (Li et al., 2013, Gao et al., 2014, Zhou et al., 2014, Karg et al., 2015), are nowadays considered the preferential nano/micro-fabrication approaches. In particular, CL allows the engineering of any structure with nano/micrometer resolution and remarkable uniformity throughout indefinitely large areas. This technique uses long-range ordered monolayers of colloidal particles as mask for further pattern transfer. Such colloids are self-assembled in honeycomb arrays whose interparticle distance can be tuned by dry-etching processes, so the final patterns maintain such periodicity. Therefore, CL naturally forms structures with a hexagonal arrangement, which allows the highest surface packing density, for a given array pitch, among other 2D lattices. This motivated the choice of the arrays of photonic elements considered here, shown in Figure 1, particularly the void structure of Figure 1B as described in the section Spheroidal Voids in TiO_2_.

In the solar cells, the only absorption (*Abs*) that generates current is that occurring in the Si layer, whereas that occurring in the other layers is *parasitic* because it corresponds to optical losses. Therefore the figure of merit in this work is the photocurrent density (*J*_*PH*_), which quantifies the full-spectrum absorption occurring in Si by integrating it, convoluted with the AM1.5 solar power spectrum (*I*_*AM1.5*_, units of Wm^−2^m^−1^), over the computed wavelength range (400–1,100 nm) (Branham et al., 2016):

(Equation 1) $$J_{PH}=e\int \frac{\text{λ}}{hc}Abs\left(\text{λ}\right)I_{AM1.5}\left(\text{λ}\right)\text{dλ}$$

where *e* is the electronic charge, *h* is the Planck constant, and *c* is the free-space light speed. This quantity is equivalent to the short-circuit current density that would be produced when electrical losses are neglected, i.e., with an internal quantum efficiency equal to 1 (IQE = 1).

An optimization algorithm (described in Section S1.3 of Supplemental Information) was used in the FDTD programs to perform a complete screening of the parameters of the LT structures (sketched in Figure 1) that yield the highest *J*_*PH*_, for three different Si absorber layers in the cells: 100- and 300-nm-thick a-Si and 1.5-μm-thick c-Si. The results of the optimizations are given in Table 1.

**Table 1:** Results of Optimized Photonic Structures

Light Trapping Structure	Material	Absorber: 100-nm a-Si		Absorber: 300-nm a-Si		Absorber: 1.5-μm c-Si		Row Label
		Optimal Parameters	*J*_*PH*_ (mA/cm^2^)	Optimal Parameters	*J*_*PH*_ (mA/cm^2^)	Optimal Parameters	*J*_*PH*_ (mA/cm^2^)	
None	No layer	–	**15.4**	–	**17.5**	–	**16.4**	1
ARC	AZO	*t* = 64.3 nm	**20.1**	*t* = 71 nm	**25.4**	*t* = 68 nm	**21.7**	2
Half-spheroids in TCO layer (Figure 1A)	TiO_2_ in AZO layer	*R* = 392.6 nm *R*_*Z*_ = 793.0 nm *d =* 863.7 nm *t* = 0 nm	**27.5**	*R* = 344.1 nm *R*_*Z*_ = 1,373 nm *d =* 985.8 nm *t* = 64.5 nm	**31.4**	*R* = 368.0 nm *R*_*Z*_ = 826.5 nm *d =* 997.3 nm *t* = 65.7 nm	**31.1**	3
Spheroidal voids in front layer (Figure 1B)	TiO_2_ layer	*R* = 449.5 nm *R*_*Z*_ = 1,517 nm *d* = 899 nm *t* = 1,060 nm	**27.0**	*R* = 429.3 nm *R*_*Z*_ = 1,007 nm *d* = 901.5 nm *t* = 1,310 nm	**32.2**	*R* = 772.9 nm *R*_*Z*_ = 1,459 nm *d =* 1,546 nm *t* = 1730 nm	**32.0**	4
	AZO layer	*R* = 375.0 nm *R*_*Z*_ = 567.1 nm *d* = 836.2 nm *t* = 666.7 nm	**23.7**	*R* = 474.9 nm *R*_*Z*_ = 1,519 nm *d* = 949.8 nm *t* = 816.7 nm	**28.1**	*R* = 588.8 nm *R*_*Z*_ = 938.2 nm *d* = 1,188 nm *t* = 987.8 nm	**26.9**	5
*Lambertian* surface	–	–	**32.4**		**33.7**	–	**38.0**	6

Maximum *J*_*PH*_ values attained for the optimized structures represented in Figure 1 placed on the three types of solar cells, with distinct Si absorber layers, considered in this work. The geometrical optimization parameters (*R, R*_*Z*_*, t, d*) are defined in Figure 1. The results are compared with the unpatterned (none) and ARC-patterned cases, as well as with the theoretical limits in the regime of geometrical optics attained with a *Lambertian* scattering surface.

Silicon remains the material of choice for PV owing to its favorable electro-optical properties, excellent reliability/durability, and high abundance (Polman et al., 2016, Vicente et al., 2017a). Thin-film Si cells are attractive due to their potential to be bendable and lightweight. As the rigidity of a layer scales with the third power of its thickness, the ultra-thin (100-nm) a-Si absorber considered here is envisaged for highly flexible devices. Such 100-nm layer can be 27 times more flexible than a layer with the conventional thickness (∼300 nm) used in standard single-junction a-Si cells (Grandidier et al., 2012, Morawiec et al., 2014, Mendes et al., 2015, Vicente et al., 2017a), which is also considered here. Another advantage of scaling down the active layer thickness, particularly in amorphous materials, is that the carrier transport loss can be significantly decreased. However, as light absorption is proportional to the film thickness, the thinner the absorber, the more challenging it becomes for LT strategies to maintain a high photocurrent. The 1.5-μm-thick c-Si absorber is motivated by the recently demonstrated production of thin (1–20 μm) mono-crystalline Si films (Yu et al., 2013, Trompoukis et al., 2015), which can combine the advantages of high material quality (e.g., high open-circuit voltage and fill factor) and thin-film technologies (e.g., low material cost, flexibility) (Bozzola et al., 2012, Andreani et al., 2015).

The present study focuses on the optical response of the materials and does not take into account their electrical quality. That is the reason why the *J*_*PH*_ values in Table 1 attained with the 300-nm a-Si absorber are generally higher than the values attained with the 1.5-μm c-Si, since in the considered spectral range the extinction coefficient (*k*) of a-Si is higher than that of c-Si (see Figure S1A). Nevertheless, here the LT performances are chiefly evaluated by the enhancement in broadband absorption, and consequent *J*_*PH*_, attained with each type of absorber relative to the reference cases without LT structures.

The results are compared with two reference cases of unpatterned cells (row 1 in Table 1, layer structure: mirror/AZO/Si) and cells patterned with a front TCO contact made of AZO, which acts as an ARC (row 2), having a thickness optimized for maximum *J*_*PH*_ with each absorber layer (Mendes et al., 2016). AZO was selected as the TCO material since optically it performs better as an ARC than a standard indium tin oxide (ITO), with the additional advantage of being composed solely of Earth-abundant materials (Lyubchyk et al., 2016a, Lyubchyk et al., 2016b, Lyubchyk et al., 2016a, Lyubchyk et al., 2016b Yin et al., 2016). The *Lambertian* light scattering case is also presented (last row in Table 1), computed with an analytical geometrical optics formalism (Bozzola et al., 2012, Branham et al., 2016, Mendes et al., 2016), which consists in an ideal anti-reflective front surface that randomizes the direction of propagation of incoming light when it enters the cell. This corresponds to the ray optics LT limit neglecting light interference effects, only valid when the cell thickness is much larger than the illuminating wavelengths. Therefore this treatment is not accurate with the present thin-film structures but is a standard comparative limit, relevant to not only analyze as benchmark but also study the physical differences arising from interference-related effects in the wave-optics regime.

### TiO_2_ Half-Spheroids Array

**Figure 2:**
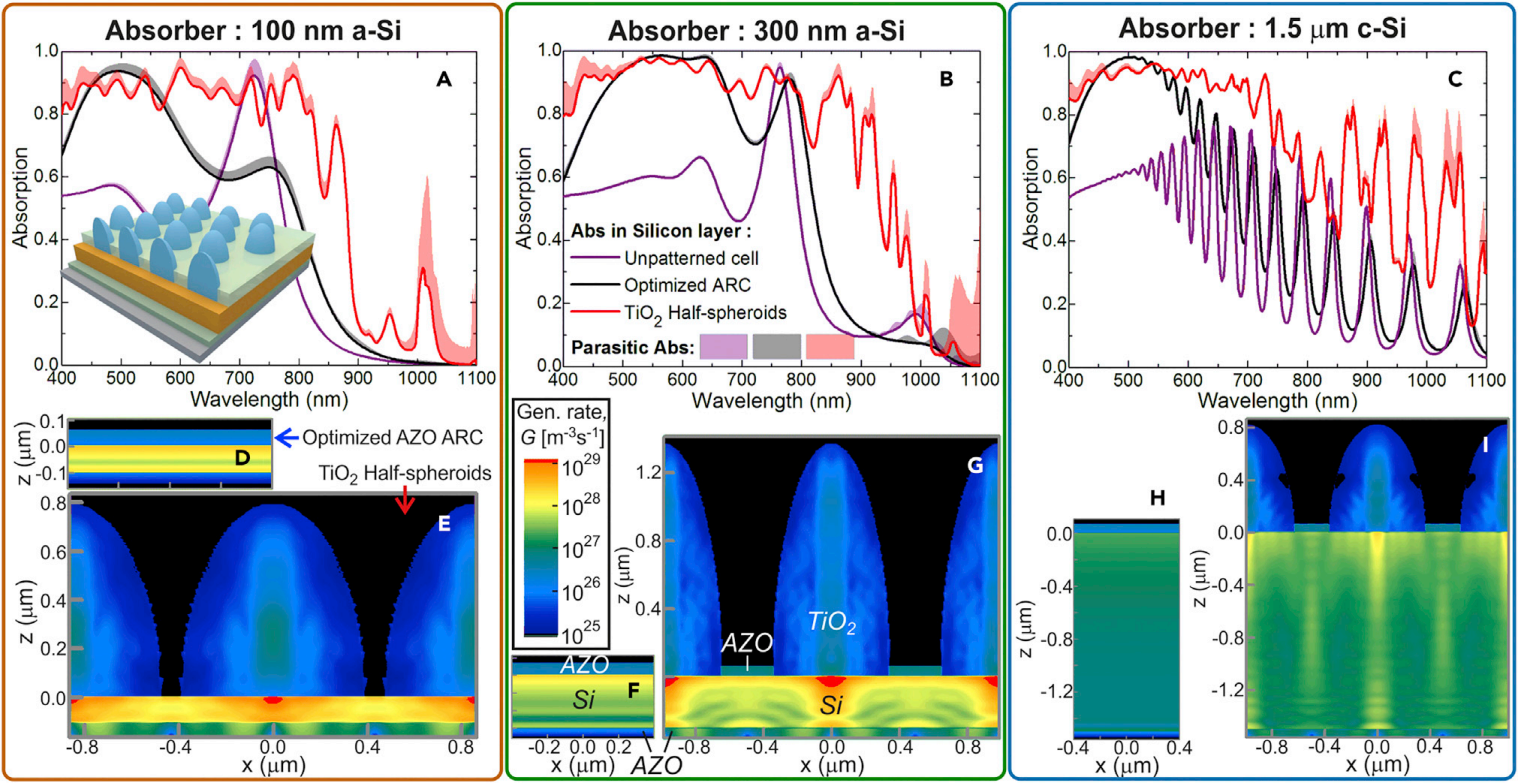
Optical Response with Optimized TiO_2_ Half-Spheroids Array (A–C) Absorption spectra attained with the optimized LT structure sketched in Figure 1A composed of half-spheroids (*red curves*, row 3 of Table 1), and with the reference cases of an unpatterned cell (*purple,* row 1) and a cell with an optimized AZO anti-reflection coating (*black,* row 2), for three absorber layers: (A) 100-nm a-Si, (B) 300-nm a-Si, and (C) 1.5-μm c-Si. Each graph shows the absorption occurring in Si (*solid lines*) and the parasitic absorption in the other materials (*colored regions above the lines*). (D–I) The bottom profiles show the log-scale distribution of the total generation rate, *G* (see Section S1.1 in Supplemental Information), along the *xz* cross-sectional plane of the structures at *y=0*. (D), (F), and (H) correspond to the reference cells with ARC, and (E), (G), and (I) correspond to those with the half-spheroids, respectively, for the 100-nm a-Si, 300-nm a-Si, and 1.5-μm c-Si absorbers.

We start by analyzing the hexagonal array of TiO_2_ half-spheroids represented in Figure 1A. The dielectric material and geometrical parameters of this LT structure have been optimized in a previous contribution (Mendes et al., 2016) for the 300-nm a-Si and 1.5-μm c-Si absorbers. TiO_2_ has been identified as one of the preferential materials, for front dielectric LT structures on thin-film cells, due to its high real part of the refractive index (*n*), which favors anti-reflection and scattering effects, and low imaginary part (*k*), which implies reduced parasitic absorption, in the relevant wavelength range (Li et al., 2013, Karg et al., 2015). Here, this study is extended to investigate the optimal LT parameters for the ultra-thin 100-nm a-Si absorber, in view of its potential for flexible cells. The results are presented in Figure 2 and compared with the reference cases of rows 1 and 2 of Table 1.

In the planar reference cells (unpatterned and ARC-patterned cells) the established electric field distributions (and thus also the generation rate, *G*, profiles) are translational invariant, exhibiting planar regions of constructive and destructive interference resulting from the light confinement between the top surface and back reflector (Yin et al., 2016). The consequent 1D *Fabry-Perot* resonances depend mainly on the Si film thickness and are responsible for the long-wavelength absorption peaks observed around 730 and 770 nm wavelengths, respectively, with the 100- and 300-nm a-Si absorbers, and the peaks for λ ≳ 550 nm with 1.5-μm c-Si. The optimized ARC layer improves considerably the absorption in the UV-Vis range, relative to unpatterned cells, but not in the NIR. Therefore it is in the NIR range (λ > 700 nm) where the LT elements become more relevant. The main reason is the pronounced scattering effects of the TiO_2_ half-spheroids, due to their optimized shape and high real part of the refractive index (*n*∼2.5–2.7), which trap the light in the high-index Si layers in two ways (Brongersma et al., 2014, Mendes et al., 2016, Yang et al., 2016): (1) the spheroids act as micro-lenses producing a strong forward-scattered near field that concentrates light in a focal region close to their bottom face, which is particularly important to confine light in the thinner a-Si absorbers, as confirmed by the intense *G* values obtained in Figures 2E and 2G and (2) for long wavelengths with path length above twice the Si layer thickness, the far-field light scattered by the spheroids is redirected to more horizontal directions along the layer, thus traversing a longer path inside Si, which can be substantially amplified when coupled with waveguide resonance modes of the structure (Spinelli and Polman, 2014, Van Lare et al., 2015). The low absorption coefficient of Si in this NIR range allows the scattered light to travel a long distance along the Si layer, suffering multiple reflections at the top and bottom surfaces and interfering with the scattered waves coming from other particles. Consequently, the hexagonal periodicity of the LT structures imposes guided modes propagating along the absorber plane that give rise to the spectrally sharp resonances observed in the NIR absorption peaks of Figures 2A–2C. These can be viewed as 3D *Fabry-Perot* resonances, in contrast with the 1D ones in the flat reference cells, as the scattered light waves inside Si interfere both in the in-plane and normal directions, which is evidenced by the dark and bright fringes of the *G* profiles of Figure 2.

At the UV-Vis wavelengths (λ < 700 nm) that do not reach the rear mirror, the absorption enhancement relative to the reference cases is mainly due to the anti-reflection action of the half-spheroids, as their shape provides geometrical effective index matching for the light traveling from air to the higher index Si layer. Similarly to the scattering effects, the optical channeling caused by index matching is favored by a high real part of the spheroids' index, as close as possible to that of Si (*n*∼4). In this wavelength range, most light is absorbed up to a depth of ∼100 nm in the Si layer, therefore the absorption spectrum attained with the LT-enhanced 100-nm a-Si cell is similar to those with the thicker layers, for λ < 700 nm. This indicates that such LT-enhanced 100-nm cell would constitute an appropriate top sub-cell for a double-junction device.

In the half-spheroids structures, the main optical role of the front AZO layer is to act as an ARC in the areas between the spheroids. Therefore the programs converged to values of its thickness (*t*, see Figure 1A) similar to those of the optimized ARC cases of row 2 of Table 1. The only exception occurred with the 100-nm a-Si absorber, since the corresponding optimized array has a pitch (*d=2.2R*) close to the spheroids' base diameter, thus establishing a close proximity between the particles and therefore, a stronger overlap of their near fields. Consequently, the anti-reflection action in this case is solely performed by the TiO_2_ half-spheroids, and the presence of an AZO layer in their narrow inter-spaces would simply reduce their optical performance. As a result, the optimization converged to a design without front AZO (*t* = 0). In practical devices, this would require the additional patterning of a top metallic network to realize the front electrical contact (Gao et al., 2014, Knight et al., 2016). Nevertheless, it was observed that there is a significant tolerance of the results with respect to *t*, as the *J*_*PH*_ values for the three different absorbers only change by ∼2% when varying *t* between 0 and 65 nm. Therefore, in the 100-nm a-Si solar cell patterned with the optimal half-spheroids array the application of a *t* = 65-nm AZO layer as the front contact would still allow a high *J*_*PH*_ of 26.9 mA/cm^2^.

### Spheroidal Voids in TiO_2_

**Figure 3:**
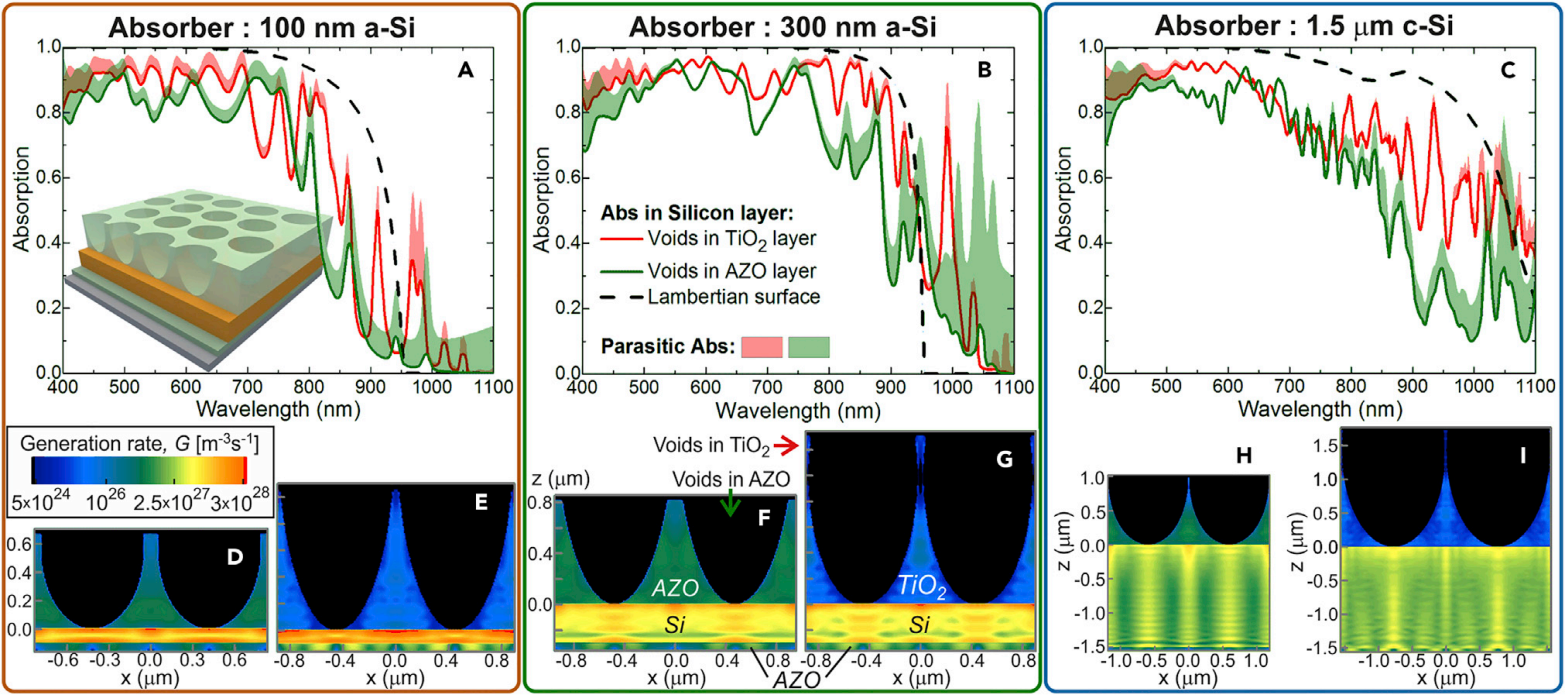
Optical Response with Optimized Spheroidal Voids Array in TiO_2_ and AZO (A–C) Absorption spectra of the LT structures with the design of Figure 1B, corresponding to rows 4 and 5 of Table 1, for the three different absorber layers: (A) 100-nm a-Si, (B) 300-nm a-Si, and (C) 1.5-μm c-Si. The absorption occurring in the Si layer (*solid lines*) and parasitic losses (*colored regions above the lines*) are shown for the semi-prolate void arrays incorporated in either a TiO_2_ (*in red*) or AZO (*in green*) front film. The spectra are compared with those (*dashed line*) calculated in the geometrical optics regime with an ideal *Lambertian* surface (row 6 of Table 1) instead of the micro-structured film. (D–I) The bottom profiles show the log-scale generation rate profiles, *G*, similarly to those of Figure 2. The profiles in (D), (F), and (H) correspond to the void arrays in an AZO film and the profiles in (E), (G), and (I) correspond to those in a TiO_2_ film, respectively, for the 100-nm a-Si, 300-nm a-Si, and 1.5-μm c-Si absorbers.

This section analyzes a novel class of front LT nanostructures, represented in Figure 1B, based on spheroidal void-like geometries, which can be viewed as the inverse design of Figure 1A. As shown in Figure 3, optimized semi-prolate voids in micrometer-thick (1–2 μm) TiO_2_ layers yield comparable LT performance as the half-prolate TiO_2_ structures of Figure 2. The *J*_*PH*_ values attained with the previous half-spheroids (row 3 of Table 1) are slightly higher (1.8%) than those with the void structures (row 4) for the 100-nm a-Si absorber, but lower than those with the void structures for the 300-nm a-Si (2.5%) and 1.5-μm c-Si absorbers (2.8%). Also, advantageously for practical implementation, the void arrays can be directly formed by CL methods consisting in four steps (Li et al., 2013, Gao et al., 2014, Karg et al., 2015, Sanchez-Sobrado et al., 2017): (1) wet coating of a close-packed monolayer of colloidal microspheres; (2) shaping the colloids with dry-etching, to form non-close-packed arrays with controllable pitch and aspect ratio of their elements; (3) deposition of the optical material (in this case TiO_2_, or AZO in the next sub-section); and (4) chemical lift-off of the colloids, leaving the nanostructured material deposited in their inter-spaces and removing that deposited above the particles. This is a versatile soft lithography method as it can pattern any material that can be deposited in the regions between the shaped colloids. Besides, it is promising for the present LT structures patterned over already completed cells, since it does not require high-temperature steps (maximum temperature ∼100°C) or other processes that can degrade the cell layers underneath.

It is noteworthy that, despite the considerable differences in the LT designs of Figures 1A and 1B, the absorption spectra (red curves in Figures 3A–3C) and generation rate profiles (Figures 3E, 3G, and 3I) of the cells with the void arrays in a TiO_2_ layer present many similarities with those of the half-spheroids of Figure 2. The overall trends of the “useful” (occurring in Si) and parasitic absorption curves are the same, but a slight improvement of the Si absorption is observed in the NIR region with the void arrays on the 300-nm a-Si and 1.5μm c-Si absorbers. The *G* profiles are also similar, but those attained with the half-spheroid arrays present more intense and localized hot spots, located close to the Si top surface beneath the particles, due to the stronger lens effect of their round shape, which creates highly intense but more confined forward-scattered near-field distributions (Mendes et al., 2011, Mendes et al., 2016, Yang et al., 2016). The cross-sectional pyramidal-like geometry, established by the void arrays in the TiO_2_ layer, provides less intense hot spots, but allows a more uniform spatial distribution of the absorbed light intensity throughout the entire Si layers. This is attributed to the NIR forward-scattering capabilities of this structure resulting in more extended/spread near-field focal regions along the absorber depth and higher optical path lengths due to improved far-field LT within the Si. A more delocalized scattered field distribution is beneficial for the thicker 300-nm a-Si and 1.5-μm c-Si absorbers, as it allows a better performance of these structures particularly in the NIR (see Figures 3B and 3C), thus explaining their slightly superior *J*_*PH*_ values (row 4 of Table 1) relative to the half-spheroid arrays (row 3). However, the thinner the absorber layer, the more advantageous the “lens effect” becomes; therefore, with the 100-nm a-Si absorber having a lower NIR response, stronger near-field focusing properties are beneficial to effectively concentrate as much light as possible in such ultra-thin active volume. This justifies the higher *J*_*PH*_ achieved in this case with the dome-like geometry of the half-spheroids array, relative to that with the voids.

The anti-reflection provided by both optimized designs of Figures 1A and 1B presents little differences, as the absorption in UV-Vis range is ∼0.9 for both types of TiO_2_ structures. This indicates that the particular dome or pyramidal-like shape of the photonic elements is not crucial to reduce reflection via index matching, and what mainly causes broadband reflection quenching is the high aspect ratio of the high-index structures (Branham et al., 2016).

The parasitic losses are represented in Figures 2A–2C and 3A–3C by the colored areas between the total absorption of the overall structures (curves not shown) and the absorption only in Si (solid lines). Figure 2 shows that the parasitic absorption in the AZO layers of the unpatterned and ARC-patterned reference cells is practically negligible, especially with the thicker absorbers. In the cells with the TiO_2_ LT structures, the parasitic absorption is more significant but still small relative to the absorption in Si. At UV-Vis wavelengths (λ < 700 nm), such optical losses occur mainly in the front TiO_2_ material; but at longer wavelengths, TiO_2_ is practically non-absorbing, so the losses chiefly take place in AZO due to the free carrier absorption of TCO materials in the NIR. Most of this absorption takes place at the rear 60-nm AZO passivating layer, present in all structures, and is boosted by the light scattering effects of the photonic elements and their coupling with the rear mirror (Mendes et al., 2016). This is demonstrated by the bright colors of the *G* distributions in the rear AZO layers of Figures 2, 3E, 3G, and 3I, relative to those of the ARC cases of Figure 2D, 2F, and 2H.

The absorption spectra in Figures 3A–3C are compared with the corresponding *Lambertian* LT case for each Si absorber, determined analytically in the geometrical optics regime (Bozzola et al., 2012, Andreani et al., 2015, Branham et al., 2016). Such so-called *Lambertian* limit is not applicable to our structures with thicknesses and photonic features below or close to the incident wavelengths, but it is, nonetheless, important to analyze as previously mentioned. Figures 2A–2C and 3A–3C show that the optimized TiO_2_ LT structures enable Si absorption values close but slightly below the *Lambertian* curves throughout most of the spectrum, with the biggest discrepancy observed for the cases with the 1.5-μm c-Si absorber, where geometrical optics predicts a quite high maximum photocurrent (38 mA/cm^2^; row 6 in Table 1), almost reaching that of bulk wafer-based cells. As the wavelength increases, the extinction coefficient (*k*) of the Si materials (see Figure S1A) becomes lower and approaches zero at the longer NIR wavelengths of below-bandgap photons, which explains the abrupt decrease of the *Lambertian* absorption curves for λ ≳ 900 nm and 1 μm, respectively, for the a-Si and c-Si absorbers. However, the omnidirectional scattering ability of the wave-optical elements and, essentially, the coupling of their scattered light with wave-guided modes trapped in the Si layer allow extending the cells' NIR response to wavelengths longer than those allowed by geometrical optics, particularly at the sharp *Fabry-Perot* resonances of the thinner a-Si structures, which generate the sharp absorption peaks for λ > 950 nm (Yu et al., 2013, Mendes et al., 2016, Yang et al., 2016).

### Spheroidal Voids in AZO

Despite the advantageous optical properties of TiO_2_ for photonic nanostructures located on the cells' front, its poor conductivity makes it difficult to engineer a good-performing front contact for the devices. With the LT structures of Figure 1B analyzed in the section Spheroidal Voids in TiO_2_, such contact could be realized by patterning a top metallic network, or micro-meshed electrodes, deposited on Si before the TiO_2_ (Gao et al., 2014, Knight et al., 2016). This is a promising approach mainly for thin c-Si active layers, as the 1.5-μm-thick one studied here. However, for a-Si cells, metallic grids alone may not be sufficient for efficient charge extraction due to the low carrier diffusion lengths in such amorphous materials (∼0.1–1 μm when compared with 0.1–1 mm in c-Si). Therefore, a good-performing front TCO layer is especially crucial for a-Si devices. As such, we now consider the same LT design of the previous sub-section (Figure 1B) but using AZO as the material of the photonic layer, instead of TiO_2_.

The abundance, non-toxicity, and excellent transparency and electrical properties of AZO cause this material to be currently regarded as the best alternative to the standard ITO transparent contacts, whose price is increasing exponentially due to the scarcity of indium (Lyubchyk et al., 2016a, Lyubchyk et al., 2016b, Lyubchyk et al., 2016a, Lyubchyk et al., 2016b, Marouf et al., 2017). Several approaches have been investigated to integrate LT structures in ZnO-based films (Battaglia et al., 2011, Despeisse et al., 2011), including growth of pyramids by chemical deposition, wet-etching of crater-like structures, and nano-texturing by imprint/molding. However, so far these approaches have been applied on the TCO front contact of thin-film Si cells with a superstrate configuration, where the Si layer is conformally deposited over the textured ZnO-based material. Although this can be optically effective, the additional roughness induced in the PV layers by the texturing degrades the devices' electrical performance, as previously described, hindering the benefits of such LT schemes. Therefore, here an innovative design is optimized wherein the wavelength-scale structured AZO is patterned on top of the flat cell layers (see Figure 1B); so the present approach is not affected by the traditional compromise between optical gains and electrical losses, as the photonic elements do not increase the devices' roughness.

The results of the optimized void-structured AZO layers are presented in Figure 3, together with those of the TiO_2_ structures with the design of Figure 1B, and the corresponding *J*_*PH*_ values are listed in row 5 of Table 1. Even though substantial photocurrent enhancements are determined with such AZO front structures, relative to the optimized ARC cases (row 2 of Table 1), they are considerably lower than those attained with TiO_2_ (row 4). This is verified by the lower Si absorption values of the solid green curves in Figures 3A–3C, relative to the red curves, throughout the analyzed spectra, in accordance with the lower values of generation rate shown in the *G* profiles of Figure 3D, 3F, and 3H along most of the Si volume. The AZO LT structures perform optically worse than the TiO_2_ ones, in terms of anti-reflection and scattering, due to the worse components of its complex refractive index (plotted in Figure S1B):

(1) Real part of refractive index (*n*): For anti-reflection via geometrical index matching, *n* should be ideally close to that of Si (*n*∼4) to favor the light incoupling from air toward such high-index absorber. Therefore, high-index dielectric materials such as TiO_2_ (*n*∼2.5–2.7) provide better impedance matching of sunlight than lower index materials like AZO (*n*∼1.8–2.0). Regarding the scattering performance affecting mainly the NIR, the higher the *n,* the higher can be the scattering cross sections of the photonic features, which lead to more intense near and far fields inside the cell absorber. Nevertheless, note that increasing *n* alone may not always guarantee photocurrent enhancement, since it amplifies the degree of interference caused by the photonic structure, which influences, for instance, the magnitude, spatial extension, and location of the light focusing beneath its features. So, it is crucial to appropriately adjust the geometrical parameters of the structures for each *n*, as also investigated in previous contributions (Mendes et al., 2010, Mendes et al., 2011, Mendes et al., 2016), to optimally tune (1) the near-field focal region to cover the depth of the underneath absorber layer, which mainly depends on the shape of the photonic elements, and (2) the far-field propagating light, in particular the waveguide mode coupling, which is chiefly influenced by the arrays' periodicity (pitch). This reveals the importance of using multi-parameter optimizations, as performed here, which take into account such dependencies by searching for the best combination of parameters that maximize the *J*_*PH*_.
(2) Imaginary part of refractive index (*k*): As seen in Figure S1B, the *k* of AZO is superior to that of TiO_2_, particularly in the NIR where it becomes three orders of magnitude higher. Therefore, the AZO LT structure provides much higher parasitic losses for λ ≳ 700 nm. This is shown in Figures 3A–3C and also in the *G* profiles (Figures 3D–3I) that present much higher values inside the front AZO structures. The relatively high *k* of this material led the optimizations to converge to smaller AZO features, with thickness values *t* considerably lower than those of the TiO_2_ structures (see rows 4 and 5 of Table 1).

In the present LT geometry, the width of the nanophotonic elements (i.e., base length of the pyramidal features viewed in the cross-sectional profiles of Figures 3D–3I) is equal to the array pitch, *d*, as sketched in Figure 1B. In general, the thicker the absorber, the more its absorption onset can be extended to longer NIR wavelengths; so such optimal width tends to increase with Si thickness to strengthen the scattering effects along the full NIR photo-response of the cells. However, increasing the features' width alone would imply decreasing their aspect ratio, which would reduce their anti-reflection effects. Therefore, the optimal layer thickness *t*, determining the features' height, must simultaneously increase to ensure the smoothest possible variation of the effective index seen by the incoming light. This explains the monotonous increase of the values of *d* and *t* (rows 4 and 5 of Table 1) with the absorber thickness, as also seen in the profiles of Figures 3D–3I.

### Angular Study

The LT structures in the previous sub-sections were optimized for illumination incident normal to the cells. Nevertheless, for practical PV applications it is important to analyze the cells' response to different incidence angles, particularly with thin-film devices that are usually not mounted on sun-tracking platforms. In addition, if the cells are flexible, they can operate in a bent state having a range of incidence angles shining throughout their active area. Therefore, if their response is not omnidirectional, a photocurrent reduction in one portion of the area can cause an overall drop in the total current supplied by the cells.

**Figure 4:**
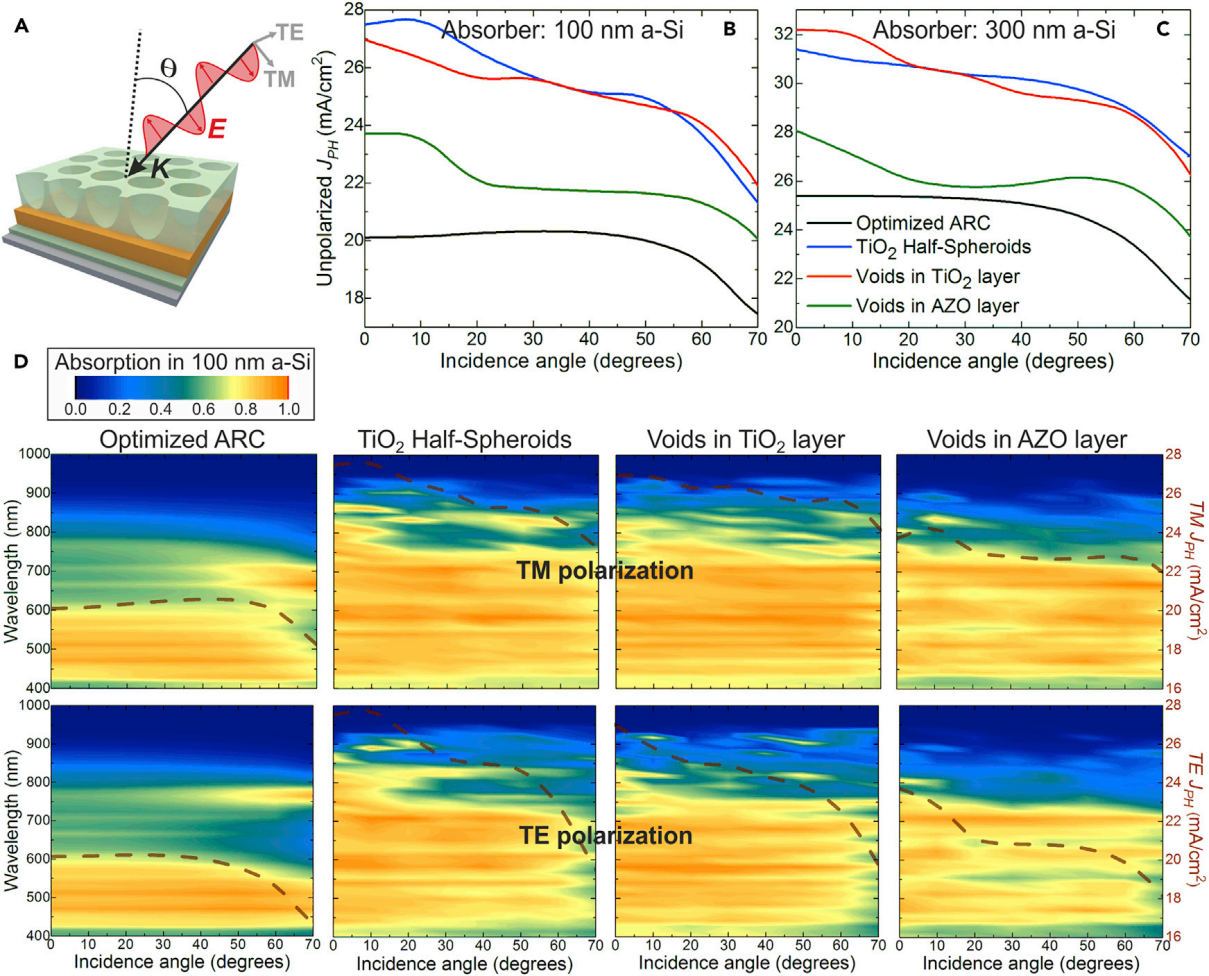
Angular Study with Optimized Photonic Structures (A) Sketch of the wave vector (***K***) and electric field (***E***) of a TM plane wave incident on the structures at an angle θ with the surface normal (*dashed line*). The ***E*** vector directions in the TM and TE components of unpolarized light are represented by the gray arrows. (B and C) Angle-resolved unpolarized photocurrent density, *J*_*PH*_, given by the average between the current values attained with the TM and TE polarizations, for the distinct optical structures analyzed in this work on the 100-nm a-Si (B) and 300-nm a-Si (C) absorber layers. (D) Contour plots of the absorption spectra occurring in the Si material of the 100-nm a-Si cells, as a function of θ, for both TM (*top*) and TE (*bottom*) polarization. The dashed brown curve plotted in the contours corresponds to the angle-resolved photocurrent density attained for each case (*values in the right axes*). See Section S2 in Supplemental Information for further details regarding the angular simulations.

In view of the above, it is advantageous to implement LT structures able to provide *J*_*PH*_ values as high and independent as possible of the incidence angle (Wilken et al., 2015). This is especially relevant for the thinner 100-nm a-Si cells, as they can be much more flexible than those with the other thicker absorbers. Therefore, here we focus on the angle-resolved optical response for the different LT structures on the ultra-thin 100-nm a-Si layer, shown in Figure 4. At oblique incidence, such response depends on the polarization of incident light, so it is important to also analyze both TM (transverse magnetic) and TE (transverse electrical) components separately (see Figures 4A and 4D). As sunlight is unpolarized, the output current is given by the average between the photocurrent values attained with each component. The angle dependence of the unpolarized *J*_*PH*_ is shown in Figure 4B and compared with that in Figure 4C attained employing the thicker 300-nm a-Si absorber layer. The FDTD computations of the angular-dependent optical response, used to determine the results of Figure 4, have been corroborated by comparison with the analytical transfer-matrix method for planar solar cell structures, as shown in Section S2 of the Supplemental Information.

Generally, the *J*_*PH*_ tends to decrease with increasing incidence angle (θ, see Figure 4A). At angles up to ∼50°, such decrease only occurs in cells with the LT structures, due to the reduction of Si absorption in the NIR as seen in Figure 4D, since the scattering and waveguide mode coupling provided by the photonic arrays are not optimized for oblique incidence. Still, with both TiO_2_ LT structures, moderate absorption peaks appear at certain angles in the NIR, caused by *Fabry-Perot* resonances excited for such illumination directions, which are less evident with the void arrays in AZO owing to the parasitic losses of this material at longer wavelengths. The general decrease of photocurrent for angles from 0 to ∼50° is not observed with the flat reference ARC structure, since it does not exhibit NIR scattering effects. For angles above ∼50°, the unpolarized *J*_*PH*_ of both the LT and ARC structures drops abruptly mainly due to the increase in reflection from the front surface (Wilken et al., 2015), explaining the decrease in the UV-Vis absorption observed in Figure 4D at such higher angles. This originates from common Brewster-type effects, causing a drop in the reflection of the TM component and an increase in the reflection of the TE component. Therefore, the TE light absorption in Si drops more abruptly at such high angles, whereas the TM absorption can even increase at certain wavelengths (Spinelli et al., 2012), as seen by the peaks in the 450–700 nm range on the right of the top contour plots of Figure 4D. Such TM peaks are the ones mainly responsible for the maxima observed in the *J*_*PH*_ curves of Figures 4B and 4C at angles in the ∼50–65° range.

## Discussion

**Figure 5:**
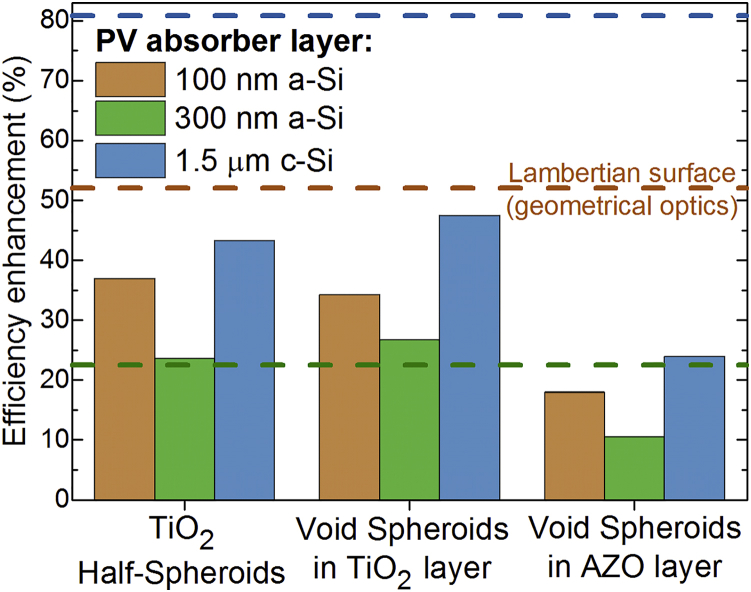
Efficiency Enhancements Anticipated with the Photonic Structures Analyzed in the Results Section, Taken to be Equal to the Photocurrent Density (*J*_*PH*_) Enhancements Relative to the Optimized ARC case (see Table 1) The horizontal dashed lines mark the enhancements attained for the theoretical *Lambertian* cases (row 6 of Table 1), considering the 100-nm a-Si (*orange line*), 300-nm a-Si (*green*), and 1.5-μm c-Si (*blue*) absorber layers. The *Lambertian* enhancement values are determined with the *J*_*PH*_ values in row 6 of Table 1, relative to the *J*_*PH*_ of the corresponding structures without light trapping, in the geometrical optics regime, given in the supplemental material of a previous contribution by Mendes et al. (2016).

The core design rule of the LT schemes investigated in this work is that they improve the cells' light harvesting without roughening their thin PV layer, in contrast with conventional texturing/structuring-based LT approaches. This was pursued by optimizing the combined anti-reflection and scattering properties of distinct front nanophotonic arrays, which do not increase the defect density (recombination) in the PV material because they are implemented over the unstructured planar absorber layer. As such, we were able to fully optimize the LT geometries toward their maximum optical performance, without concerns relative to the devices' electrical performance. Therefore, the efficiency gains resulting from the LT structures presented here are expected to be similar to their corresponding *J*_*PH*_ enhancements. The bar graph in Figure 5 shows such predicted gains, relative to the optimized ARC case of row 2 of Table 1, and compares them with those analytically determined in the *Lambertian* case of geometrical optics (row 6 of Table 1). Note that the *Lambertian* enhancements were not calculated relative to the same reference values (ARC cases of Table 1) of the optimized LT structures, but rather relative to the same theoretical structure but without light scattering from the front surface (Bozzola et al., 2012, Mendes et al., 2016).

For the three types of LT nanostructures explored in this work, the highest photocurrent enhancements are attained with the 1.5-μm c-Si absorber and the lowest are attained with the 300-nm a-Si. Such trend follows that predicted for the *Lambertian* cases, shown by the dashed lines of Figure 5 and analyzed in further detail in previous contributions (Bozzola et al., 2012, Callahan et al., 2012, Mendes et al., 2016). The highest enhancement values achieved with 1.5-μm c-Si are because c-Si is an indirect bandgap semiconductor with a lower extinction coefficient in most of the analyzed spectral range (see Figure S1A) than a-Si, which is a more absorbing direct semiconductor. Therefore, thin c-Si cells can benefit more from LT than a-Si ones, since c-Si requires higher dwelling time (path length) of the photons in the active region, as provided by scattering, to complete the absorption of the full range of above-bandgap photons before they escape the device (Andreani et al., 2015, Trompoukis et al., 2015). On the other hand, generally LT also becomes more beneficial with decreasing cell thickness, since the thinner the Si layer, the more pronounced can be the absorption enhancement, as analytically computed with geometrical optics. Consequently, higher enhancements were obtained with the 100-nm a-Si absorber relative to the 300-nm a-Si. Nevertheless, as seen in Figure 5, the 300-nm a-Si absorber reached enhancements above that of the corresponding *Lambertian* case (22.5%), particularly with the TiO_2_ voids. This is because such LT structure produced pronounced absorption peaks at NIR wavelengths (λ > 950 nm) above the onset of the *Lambertian* absorption spectrum (see Figure 3B), which originate from wave-optical interference effects resulting from the coupling of the scattered light with guided modes trapped by multiple reflections in the cell (Callahan et al., 2012). However, it should be noted that the absolute values of the maximum *J*_*PH*_ given in Table 1 are below those of the *Lambertian* limits (row 6) for all the LT structures optimized here.

The TiO_2_ void structure introduced in this work allowed the highest enhancements (26.4% and 47.5%, respectively) for the 300-nm a-Si and 1.5-μm c-Si absorbers, as its cross-sectional pyramidal shape provides effective anti-reflection at short (UV-Vis) wavelengths and a better coverage of the forward-scattered field throughout such Si layers, relative to the TiO_2_ half-spheroids. The dome shape of the half-spheroids provides a higher optical lens effect, which results in a stronger localization of the forward-scattered light. Such effect is, however, advantageous for light confinement in ultra-thin absorber layers, as demonstrated by the highest current enhancement (37.0%) attained with the 100-nm a-Si absorber topped with the half-spheroids array.

The lowest enhancements in Figure 5 were obtained with the AZO void arrays due to the weaker anti-reflection and scattering effects allowed by this material, as a consequence of its lower real part of the refractive index relative to TiO_2_ and its higher parasitic losses mainly in the NIR range (see Figure S1B). Despite such more modest enhancements (11–24%), this AZO LT structure offers an unprecedented practical advantage: it constitutes the TCO front electrode of the cell, allowing the use of higher TCO volume (hence yielding lower contact resistance) relative to that employed conventionally. The volume of front AZO (*V*_*AZO*_) in the LT structure of Figure 1B is determined by subtracting the volume of the semi-prolate holes from the total parallelepiped layer volume. Dividing the resulting AZO volume by its covered area (*A*_*AZO*_) yields a volume-equivalent layer thickness (*t*_*eq*_ = *V*_*AZO*_*/A*_*AZO*_) equal to 319, 490, and 387 nm, respectively, for the AZO LT structures on the 100-nm and 300-nm a-Si and 1.5-μm c-Si cells. Such *t*_*eq*_ values are considerably higher than the layer thicknesses used in conventional flat TCO front contacts, which typically range from 60–80 nm (as in the reference ARC cases considered here) to 200–250 nm in *n-i-p* thin-film Si cells (Grandidier et al., 2012, Morawiec et al., 2014, Águas et al., 2015, Mendes et al., 2015), depending on the TCO material. Higher layer thicknesses lead to detrimental optical losses with flat TCOs, but not with photonic-structured TCOs as those optimized here. Therefore, the AZO void structure can provide, in practice, higher efficiency enhancements than those predicted in Figure 5, as it may increase not only the generated current but also the cells' fill factor due to the reduction of series resistance of the front contact.

### 3.1 Comparison with Other Light Trapping (LT) Approaches

As the main role of LT is the improvement of light absorption in the PV layer, the LT performance is commonly evaluated by the photocurrent enhancement achieved relative to planar reference solar cells without the LT structures but with an optimized single-layer ARC, as performed in this work.

In commercial wafer-based c-Si cells the texturing is applied on the front surface of the absorber, so this approach is difficult to implement in thin-film cells since the dimensions of the texturing features (≳ 1 μm) (Ingenito et al., 2014) are usually higher or comparable to the cells' thickness. Nevertheless, when applying optimized texturing on the front of thin (5 μm) c-Si wafers a maximum enhancement of 37% has been attained (Branham et al., 2016). In thin-film Si cells with an *n-i-p* layer configuration, compatible with opaque substrates, LT is commonly based on texturing the cells' substrate. This is performed, for instance, by employing the Asahi standard textured substrate widely used in industrial devices, which has been shown to provide about 20% and 37% photocurrent enhancement in conventional 350-nm a-Si (Van Lare et al., 2015) and 1.5-μm μc-Si (Mendes et al., 2015) cells, respectively. When the morphology of the substrate corrugations is controlled with lithographic processes, for example, employing a 2D photonic grating of dielectric nanocavities, higher enhancements can be reached (23% in 350-nm a-Si) (Van Lare et al., 2015).

Alternative wave-optical solutions, based in front-located dielectric elements on planar solar cells, have mainly considered self-assembled monolayers of spherical colloids (e.g., silica [Grandidier et al., 2012] or TiO_2_ [Yang et al., 2016] patterned on 100-nm a-Si cells). Despite the fabrication simplicity, the silica particles allowed only modest 10%–15% current enhancements due to their low refractive index, whereas with the TiO_2_ particles, a much higher enhancement of 43% is modeled but relative to a non-optimized 80-nm ITO ARC. Superior performances can be attained with higher aspect ratio dome/pyramidal-like geometries, mainly due to the better geometrical index matching that they provide for broadband anti-reflection, which is the class of structures optimized in this work. Sets of this type of TiO_2_ LT front structures with close-packed features have been optically modeled (Tseng et al., 2012), yielding a maximum 33% photocurrent enhancement in 2-μm c-Si cells.

The best *J*_*PH*_ enhancements indicated in Figure 5 for the TiO_2_ LT structures (37%, 27%, and 48% in 100-nm a-Si, 300-nm a-Si, and 1.5-μm c-Si, respectively) are among the highest attained so far in the literature, when comparing the values for similar Si absorbers. Apart from the favorable choice of geometries and material of the LT elements investigated here, our superior results are chiefly attributed to a complete multi-variable optimization process (see Section S1.3 in Supplemental Information) that explored the full domains of the main parameters of the photonic structures, in contrast with most related studies limited to certain parameter sets or to sequential parameter sweeps (Tseng et al., 2012). When more than two parameters are involved, such sweeps are not effective in finding the global maximum of figures of merit of complex systems that have a strong interdependence between the parameters, as in the present regime of wave-optics. Therefore, the multi-parameter optimizations performed in this work enabled the design of LT-enhanced cells capable of producing photocurrent density values (up to 27.5, 32.2, and 32.0 mA/cm^2^ in 100- and 300-nm a-Si and 1.5-μm c-Si, respectively) approaching those (32.9 mA/cm^2^) of the present record thin-film (∼4 μm thickness) Si single-junction cell, fabricated on an optimized honeycomb-textured substrate but using a much thicker microcrystalline Si layer (Sai et al., 2015). As the LT structures developed here do not compromise the cells' electrical performance, they can thereby allow PV devices with improved efficiencies using thinner absorber layers, which enable additional advantages such as lower material costs and mechanical bendability.

### Angular Dependence of Light Collection Enhancement

**Figure 6:**
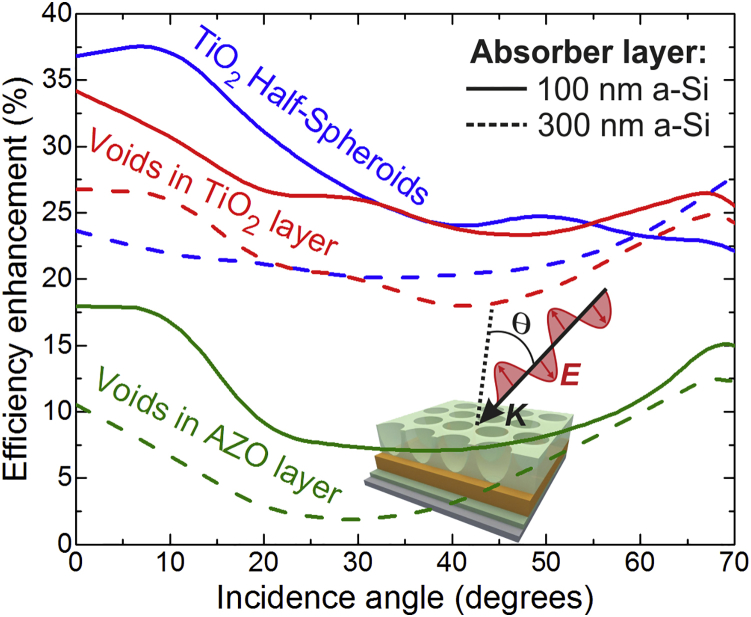
Predicted Efficiency Enhancement for the Three LT Structures Analyzed in This Work, Relative to the ARC Reference Case, as a Function of Incidence Angle The curves are determined with the *J*_*PH*_ values of Figures 4B and 4C, considering the LT structures on the 100-nm a-Si (*solid lines*) and 300-nm a-Si (*dashed lines*) absorber layers, respectively.

Regarding the angular dependence analysis (section Angular Study), whereas the *J*_*PH*_ attained with the optimized ARC is approximately maintained from 0 to ∼50°, that with the LT structures drops by about 15%–20% in this angular range due to the weakening of the scattering effects. Nevertheless, the *J*_*PH*_ provided by the LT structures remains above that of the ARC case for the entire 0–70° angular range, as shown by the enhancement curves in Figure 6. In general, such enhancement tends to decrease with increasing angle up to ∼40°, but then increases for higher angles due to the reduction of the anti-reflection effects, which causes a more abrupt photocurrent drop for the ARC case in this higher angle range than for the LT cases. In the continuation of the values in Figure 5 for normal incidence, the enhancements attained in the 100-nm a-Si cells remain above those with the 300-nm a-Si absorber, for practically all angles. However, the separation between the curves of these absorbers becomes smaller with increasing angle, which is expected as light impinges on the structures from a more horizontal (in-plane) direction that is less sensitive to the cell thickness.

### Conclusions

The goal of obtaining high-efficiency (>20%), inexpensive (<1$/W), and flexible (bending radius ≲ 5 mm) solar cells, amenable to industrial implementation, is currently one of the hottest research topics in PV wherein light management has shown to be indispensable. The findings presented here are an essential contribution in this context, to realize ever-thin cells with optimized light harvesting and electronic transport.

Two classes of front-located LT nanostructures, composed of wavelength-sized features, were computationally designed to maximize absorption in the underlying PV layer without degrading the devices' electrical performance. The optical roles of such structures have been demonstrated by (1) drastically reducing reflection losses at short wavelengths (above the absorber bandgap) via geometrical refractive index matching with the cell media and (2) enhanced absorption of longer wavelengths by increasing their path length via light bending and coupling with wave-guided modes confined in the absorber layer. These combined effects provide broadband absorption enhancement, which allows reducing the absorber thickness without lowering its output current. Here, it is important to underline that our results demonstrate that such optical effects perform remarkably well with LT structures having quite distinct designs, as those investigated (see Figure 1), as long as their parameters are fully optimized. This is quite relevant, especially for industrial implementation, as it yields more versatility in the choice of the photonic structures, enabling the integration of the design that is more convenient for large-scale fabrication.

Although the structures have been optimized for absorption enhancement in thin-film Si cells, the insights gained are generic and can be straightforward applied to any type of thin-film PV devices, with distinct materials or thickness, by carefully tuning the physical parameters analyzed here.

## Methods

All methods can be found in the accompanying Transparent Methods supplemental file.

## Supplemental Information

Document S1. Transparent Methods and Figures S1 and S2
