# Supplementary material for: Optimal-Enhanced Solar Cell Ultra-thinning with Broadband Nanophotonic Light Capture
Source: iScience. 2018 Apr 26;3:238–54. doi: 10.1016/j.isci.2018.04.018 (PMC6137392; doi:10.1016/j.isci.2018.04.018)
Supplement: Document S1. Transparent Methods and Figures S1 and S2 [file mmc1.pdf]

ISCI, Volume *3*

Supplemental Information

Optimal-Enhanced Solar Cell Ultra-thinning with Broadband Nanophotonic Light Capture

Manuel J. Mendes, Sirazul Haque, Olalla Sanchez-Sobrado, Andreia Araújo, Hugo Águas, Elvira Fortunato, and Rodrigo Martins

Supplemental Information

S1 – Transparent Methods

S1.1 – Numerical model

The formalism used to model the optical effects of the wavelength-sized light trapping (LT) structures in thin-film solar cells is based in a 3D finite-difference time domain (FDTD) numerical method (*Lumerical Solutions, Inc.*, 2017). The method solves Maxwell's equations in arbitrary geometries and materials, being one of the preferential approaches for electromagnetic problems in the wave-optics regime, particularly for light management in PV devices. For the photonic structures and solar cell architectures considered in this work (shown in Fig. 1), the FDTD simulations were performed with a mesh design adapted to the structures, composed of spatial step sizes limited between 0.25-5 nm. The maximum physical FDTD time imposed to the simulations was 1500-2000 fs, with a time step of 0.015 fs. According to extensive validation tests performed by the authors, these computational settings (and those described in the next paragraphs) revealed to be sufficient for the accurate convergence of the electromagnetic field solutions in the investigated structures (Mendes *et al.*, 2016).

The complex refractive-index functions of the materials considered in the FDTD programs were taken from standard experimentally-measured spectra (Palik, 1997; *Refractive Index Database*, 2017), plotted in the following sub-section S1.2. The hexagonal periodicity of the structures allows the FDTD region to be reduced to the rectangular unit cell represented in the top views of Fig. 1. Specific boundary conditions (BCs) are applied in each face of such rectangular box. A perfect-matching layer is applied on the upper boundary (z_{MAX}) to absorb all outgoing waves. A perfect metallic BC is used on the front surface of the rear mirror (z_{MIN}) to simulate a perfect reflecting layer, which assists in LT as the transmitted light is returned to the cell without optical losses. On the side boundaries (*x,y*), periodic BCs are used to model the infinite periodicity of the structures. Here, due to the symmetries of the system at normal incidence, symmetric and anti-symmetric BCs are employed which allow simulating only one quadrant (red region in top views of Fig. 1) of the unit cell. However, such symmetry does not hold for oblique incidence. Therefore, in the angular studies of section 2.4, the simulation volume was extended to the entire rectangular unit cell and periodic Bloch BCs were used in the *(x,y)* boundaries. In section S2 below, validation results for different incidence angles and polarizations are presented, demonstrating a precise match between the FDTD-computed absorption spectra of cell structures with those calculated analytically with the transfer-matrix method.

To simulate solar illumination, a broadband plane-wave source is placed in air above the structure. Each simulation is performed for 301 wavelength (λ) points considered at equally spaced wavelengths along the 400-1100 nm source wavelength range, since the AM1.5 solar photon flux outside this bandwidth is small, and it corresponds to the most significant portion of the photocurrent spectrum of Si-based cells. At a certain illumination angular frequency (ω), the power absorbed per unit volume ($P_{ABS}(\omega)=\omega\varepsilon''|\boldsymbol{E}|^2/2$) in each element of the structures is given by the resulting electric-field ($\boldsymbol{E}$) distribution established in their material; where ε'' is the imaginary part of the dielectric permittivity. The number of photons absorbed per unit volume and per unit time is $g(\omega)=P_{ABS}/E_{PH}$; where $E_{PH}=\hbar\omega$ is the photon energy. Here we assume that each absorbed photon excites one electron-hole pair; so the photon absorption rate, *g*, is equivalent to the optical generation rate. When considering a range of illumination frequencies, as in the present case of a broadband source characterized by a spectral irradiance (instead of a power density), the E-field is replaced by an electric-field spectral density such that its intensity, $|\boldsymbol{E}|^2$, becomes with units of $V^2m^{-2}Hz^{-1}$. In this way, *g* is generalized to a spectral generation rate (units of $m^{-3}s^{-1}Hz^{-1}$) such that the total generation rate (*G*, units of $m^{-3}s^{-1}$) can be calculated by integrating over the frequency (*Lumerical Solutions, Inc.*, 2017): $G=\int g(\omega)d\omega$.

In the solar cells, the only absorption (*Abs*) that generates current is that occurring in the Si layer, while that occurring in the other layers is *parasitic* as it corresponds to optical losses. Therefore, the figure-of-merit in this work is the photocurrent density (J_{PH}) which quantifies the full-spectrum absorption occurring in Si by integrating it, convoluted with the AM1.5 solar power spectrum ($I_{AM1.5}$, units of $Wm^{-2}m^{-1}$), over the computed wavelength range (400-1100 nm) (Branham *et al.*, 2016):

$$J_{PH} = e\int \frac{\lambda}{hc} Abs(\lambda)\, I_{AM1.5}(\lambda)\mathrm{d}\lambda \qquad (1)$$

where e is the electronic charge, h the Planck constant and c the free-space light speed. This quantity is equivalent to the short-circuit current density that would be produced when electrical losses are neglected; i.e. with an internal quantum efficiency equal to one (IQE=1).

S1.2 – Complex refractive index spectra

The real (n) and imaginary (k) components of the complex refractive index functions of the materials considered in the FDTD programs were taken from common experimentally-measured spectra, available in the literature (Palik, 1998) and in an online repository (*Refractive Index Database*, 2017), which are shown in Fig. S1.

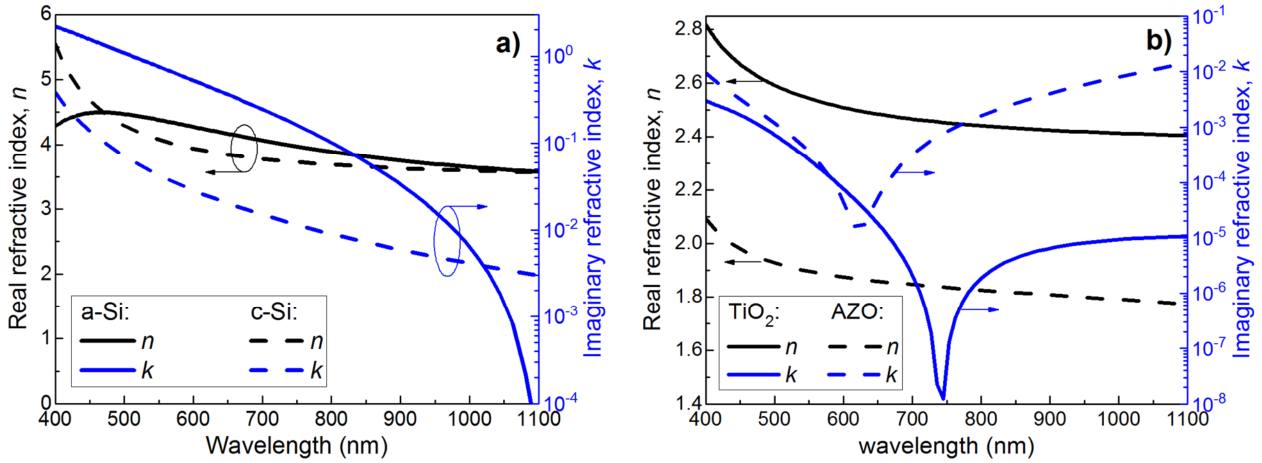

Figure S1, related to Fig. 1 and Table 1: Real (*black curves, left axis*) and Imaginary (*blue curves, right axis*) parts of the refractive index of: **a)** silicon materials - amorphous (a-Si, *solid lines*) and crystalline (c-Si, *dashed lines*), and **b)** dielectric (TiO_2, *solid lines*) and transparent conductive oxide (AZO, *dashed lines*) materials.

S1.3 – Optimization algorithm

A *particle swarm* optimization (PSO) algorithm (*Lumerical Solutions, Inc.*, 2017) was used in the programs to perform a complete screening of the parameters of the LT structures (sketched in Fig. 1) that yield the highest J_{PH}. The algorithm iteratively adjusts the structures' geometry to maximize absorption in Si, while minimizing optical losses (i.e. total reflection and absorption occurring in AZO and/or TiO_2 materials). Population-based stochastic optimization techniques as PSO are preferential when operating with complex physical systems, as in the present wave-optics regime where there is a strong correlation between all parameters (Mendes, Schmidt and Pasquali, 2008), making it practically unfeasible to accurately determine the maximum of any figure-of-merit by sequential parameter sweeping (Yang *et al.*, 2016; Vicente *et al.*, 2017). The geometrical parameters of the LT structures represented in Fig. 1 and listed in Table 1 were taken as variables to maximize the J_{PH} [Equation (1)] using the PSO algorithm. The domain of each variable was restricted to avoid searching in physically forbidden regions or where it is known a priori that the figure-of-merit is low. Here, the domains imposed on the variables were: $0 < R$, R_Z, $t < 2$ μm and $2R < d < 4R$.

In each optimization step, the basic procedure to maximize a function with an arbitrary number of variables is to randomly pick a set of starting points (called the population size) within the domains defined for such variables, at which the function is evaluated. The algorithm then iteratively moves and redimensions the set of points along the coordinate space as better points are found until some desired bound is obtained. However, this can correspond to a local maximum in the search domain. To find the global maximum, several optimization steps are sequentially run with different initial sets of points within the domain. In the optimizations performed in this work, the population sizes were in the range of 20-25, the number of iterations required per optimization step to reach a maximum was about 20-35, and usually the global maximum was found within 10-15 optimization steps with different starting populations.

S2 – Corroboration of angular simulations

The numerical simulations of section 2.4 considering oblique incidence (see Fig. 4) are more computationally challenging than those for normal incidence, since they require higher mesh resolution, longer FDTD time and the definition of periodic boundary conditions (BCs) of the Bloch type, in the in-plane boundaries of the structures' unit cell, which differ from the symmetric and anti-symmetric BCs usually employed for normal incidence on periodic structures (see Fig. 1) (Mendes *et al.*, 2016; *Lumerical Solutions, Inc.*, 2017). The mesh resolution was adapted to the structures geometry and illumination conditions, after sets of convergence tests, to minimize the computational requirements while maintaining high accuracy. Several validation tests have been performed to verify the accuracy of the computations along the range of incidence angles (0-70º) considered in this work. Figure S2 shows some of such results where the total absorption spectra determined numerically by FDTD is shown to precisely match those calculated analytically using the well-known transfer-matrix method, for different angles and polarizations (TM and TE) of the impinging light. Such comparison is only possible for flat cell structures, as that of the reference ARC case (row 2 of Table 1), since the analytical transfer-matrix formalism is solely applicable to multi-layered film structures without scattering phenomena.

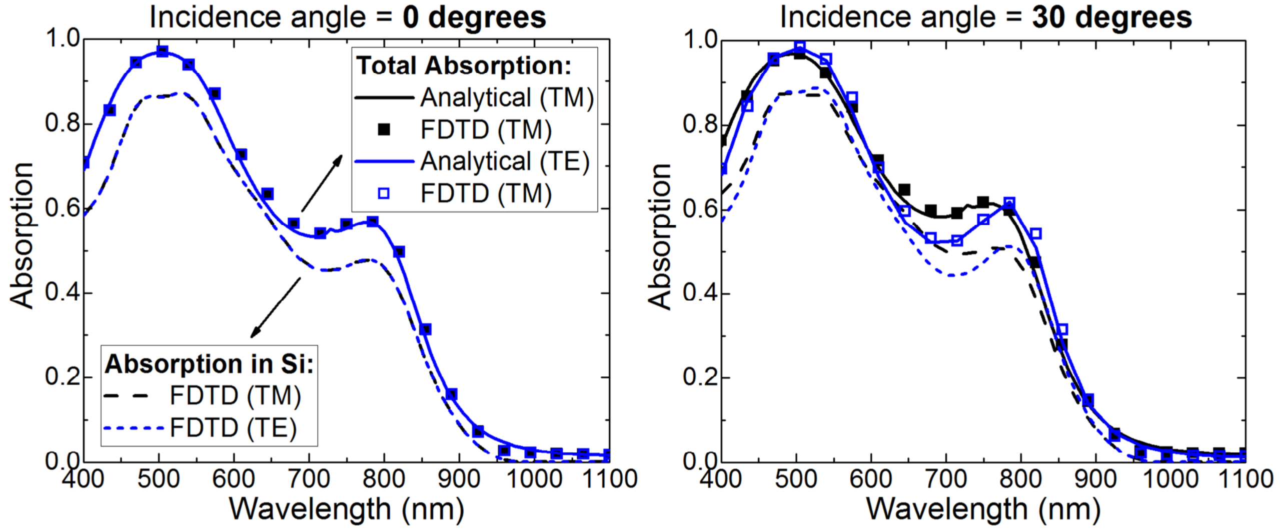

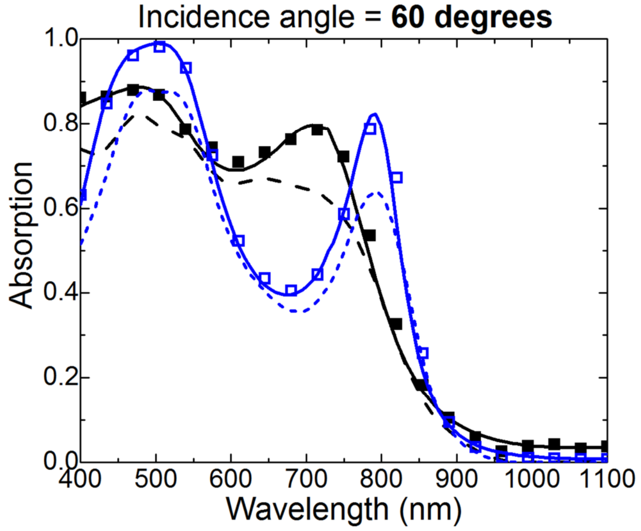

Figure S2, related to Fig. 4: Comparison between the total absorption calculated analytically with the transfer-matrix method (*solid lines*) and that computed numerically with FDTD (*square symbols*), for a flat cell structure: Ag mirror (120 nm)/AZO (60 nm)/a-Si (100 nm)/AZO (64.3 nm). Each graph represents the absorption occurring in the entire cell structure and that occurring only in the a-Si layer (*dashed lines*), for illumination with both TM (*in black*) and TE (*in blue*) polarization and three different incidence angles: 0º, 30º and 60º.

References

Branham, M. S. *et al.* (2016) 'Empirical Comparison of Random and Periodic Surface Light-Trapping Structures for Ultrathin Silicon Photovoltaics', *Advanced Optical Materials*, 4(6), pp. 858–863. doi: 10.1002/adom.201500667.

Lumerical Solutions, Inc. (2017) *Lumerical Solutions, Inc.* Available at: www.lumerical.com (Accessed: 1

February 2018).

Mendes, M. J. *et al.* (2016) ‘Design of optimized wave-optical spheroidal nanostructures for photonic-enhanced solar cells’, *Nano Energy*. Elsevier, 26, pp. 286–296. doi: 10.1016/j.nanoen.2016.05.038.

Mendes, M. J., Schmidt, H. K. and Pasquali, M. (2008) ‘Brownian Dynamics Simulations of Single-Wall Carbon Nanotube Separation by Type Using Dielectrophoresis’, *The Journal of Physical Chemistry B*. American Chemical Society, 112(25), pp. 7467–7477. doi: 10.1021/jp711450w.

Palik, E. D. (1997) ‘Handbook of Optical Constants of Solids’, in *Academic Press*. San Diego, CA. doi: 10.1016/B978-012544415-6.50097-2.

Palik, E. D. (1998) *Handbook of optical constants of solids*. Academic Press.

Refractive Index Database (2017). Available at: http://refractiveindex.info (Accessed: 1 February 2017).

Vicente, A. T. *et al.* (2017) ‘A statistics modeling approach for the optimization of thin film photovoltaic devices’, *Solar Energy*, 144, pp. 232–243. doi: 10.1016/j.solener.2017.01.029.

Yang, Z. *et al.* (2016) ‘Scattering effect of the high-index dielectric nanospheres for high performance hydrogenated amorphous silicon thin-film solar cells’, *Scientific Reports*. Nature Publishing Group, 6, p. 30503. doi: 10.1038/srep30503.
